# Transcriptomic profiling of hepatic tissues for drug metabolism genes in nonalcoholic fatty liver disease: A study of human and animals

**DOI:** 10.3389/fendo.2022.1034494

**Published:** 2023-01-04

**Authors:** Li Chen, Lu Chen, Xu Li, Lin Qin, Yan Zhu, Qianru Zhang, Daopeng Tan, Yuqi He, Yu-He Wang

**Affiliations:** ^1^ Department of Pharmacy, Affiliated Hospital of Zunyi Medical University, Zunyi, China; ^2^ The Key Laboratory of the Ministry of Education of the Basic Pharmacology, School of Pharmacy, Zunyi Medical University, Zunyi, China; ^3^ The Joint International Research Laboratory of Ethnomedicine of the Ministry of Education, School of Pharmacy, Zunyi Medical University, Zunyi, China

**Keywords:** nonalcoholic fatty liver disease, transcriptome, drug metabolism genes, GEO datasets, high-fat diet

## Abstract

**Background:**

Drug metabolism genes are involved in the *in vivo* metabolic processing of drugs. In previous research, we found that a high-fat diet affected the transcript levels of mouse hepatic genes responsible for drug metabolism.

**Aims:**

Our research intends to discover the drug metabolism genes that are dysregulated at the transcriptome level in nonalcoholic fatty liver disease (NAFLD).

**Methods:**

We analyzed the transcriptome for drug metabolism genes of 35 human liver tissues obtained during laparoscopic cholecystectomy. Additionally, we imported transcriptome data from mice fed a high-fat diet in previous research and two open-access Gene Expression Omnibus (GEO) datasets (GSE63067 and GSE89632). Then, using quantitative real-time polymerase chain reaction (qRT-PCR), we cross-linked the differentially expressed genes (DEGs) in clinical and animal samples and validated the common genes.

**Results:**

In this study, we identified 35 DEGs, of which 33 were up-regulated and two were down-regulated. Moreover, we found 71 DEGs (39 up- and 32 down-regulated), 276 DEGs (157 up- and 119 down-regulated), and 158 DEGs (117 up- and 41 down-regulated) in the GSE63067, GSE89632, and high-fat diet mice, respectively. Of the 35 DEGs, nine co-regulated DEGs were found in the Venn diagram (*CYP20A1*, *CYP2U1*, *SLC9A6*, *SLC26A6*, *SLC31A1*, *SLC46A1*, *SLC46A3*, *SULT1B1*, and *UGT2A3).*

**Conclusion:**

Nine significant drug metabolism genes were identified in NAFLD. Future research should investigate the impacts of these genes on drug dose adjustment in patients with NAFLD.

**Clinical Trial Registration:**

http://www.chictr.org.cn, identifier ChiCTR2100041714.

## 1 Background

Drug metabolism refers to a series of organic reactions that occur after a drug enters the body. The process is separated into three phases, mostly involving phase I metabolic reactions, phase II metabolic reactions, and drug transport processes. Phase I metabolism consists of functionalization reactions, phase II drug metabolism is a coupling reaction, and phase III refers to transporter-mediated clearance of drugs and/or metabolites from the body. These processes often occur through the liver, intestines, kidneys, or lungs. Hundreds of drug metabolism genes are involved in these mechanisms. There are numerous drug metabolism gene families, including alcohol dehydrogenase (ADH), aldehyde dehydrogenase (ALDH), cytochrome P450 (CYP), flavin-containing monooxygenase (FMO), monoamine oxidase (MAO), carboxylesterases (CESs), methyltransferases (METs), N-acetyltransferases (NATs), uridine 5’-diphospho-glucuronosyltransferases (UGTs), glutathione S-transferases (GSTs), sulfotransferases (SULTs), dihydropyrimidine dehydrogenases (DPDs), thiopurine methyltransferases (TPMTs), catechol O-methyltransferases (COMTs), ATP-binding cassette (ABC) transporters, solute carrier (SLC) transporters and so on (1, 2). If the expression levels of drug metabolism genes change in different physiological and pathological situations, it potentially affects the blood/plasma clearance of drugs eliminated by hepatic metabolism or biliary excretion, it can also affect plasma protein binding, which in turn could influence the processes of distribution and elimination (3). Thus, understanding the alterations of drug metabolism genes in various pathological statuses is the key step to guaranteeing the precision of the therapeutic effect.

Depending on the severity of the condition, nonalcoholic fatty liver disease (NAFLD), a common liver disease, is divided into simple steatosis (SS) and nonalcoholic steatohepatitis (NASH). With a global prevalence of approximately 25%, NAFLD is strongly associated with cardiovascular disease and metabolic diseases such as obesity and type 2 diabetes. However, therapeutic advances have been slow. NAFLD has several negative effects, including the development of end-stage liver disease and cancer. Liver transplantation is always necessary for end-stage liver disease. Globally, it is estimated that 20 million people will eventually die of NAFLD-related liver disease, while the risk of type 2 diabetes and cardiovascular disease also increases. Hence, the burden of NAFLD has become a major public health issue (4–6). The dysregulation of drug metabolism genes in NAFLD has been reported in several studies. For instance, a high-fat diet-induced NAFLD in male C57BL/6 mice. During the development of hepatic steatosis (8–16 weeks), the expression of genes *Cyp3a11*, *Cyp2b10*, *Abcg5*, and *Abcg8* was significantly elevated (7). Jiao et al. (8) found that the expression of bile acid synthesis enzymes (CYP7A1, CYP8B1, and CYP27A1) and two transporter proteins (OATP1B1 and OATP1B3) were increased in NASH patients. However, the relationship between NAFLD and drug metabolism genes cannot be fully and systematically understood by individual gene-level investigations. Traditional study designs have insufficient sample sizes and limit the scope and depth of studies.

Transcriptomics has paved the way for a comprehensive understanding of how genes are expressed and interconnected. Drug metabolism genes associated with NAFLD have not been fully characterized and are an active research area. In previous research, we investigated the transcriptome of mouse hepatic 612 drug metabolism genes to profile the changes resulting from a high-fat diet (9). A total of 476 genes were found to be homologous to Homo sapiens. In the current study, we used RNA sequencing technology to investigate these 476 drug metabolism genes in healthy controls (HC) and NAFLD liver tissues to profile NAFLD-induced changes in human liver samples. However, solid results may be difficult to obtain due to the small sample size and false-positive rate in single transcriptome analysis. Thus, two microarray datasets obtained from Gene Expression Omnibus (GEO) were utilized for the analysis. We anticipate that the results of the current investigation will reveal the drug metabolism genes which are dysregulated at the transcriptome level in NAFLD.

## 2 Materials and methods

### 2.1 Study design

**Figure 1:**
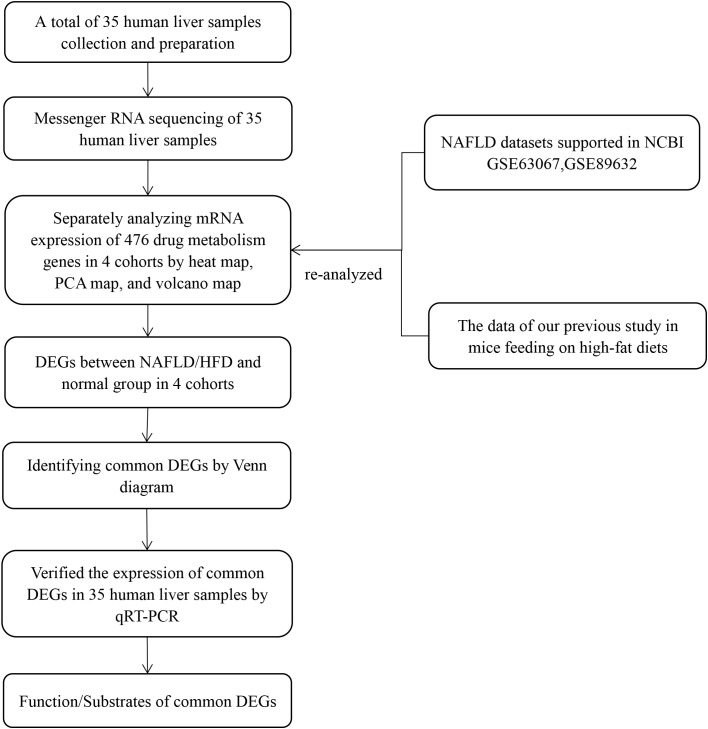
Flow diagram of the study design. The four cohorts were the human liver samples collected, the GEO dataset GSE63067, the GEO dataset GSE89632, and our previous mouse data. PCA, principal component analyses; NAFLD, nonalcoholic fatty liver disease; HFD, high-fat diet; DEGs, differentially expressed genes; qRT-PCR, quantitative real-time polymerase chain reaction.

We created inclusion and exclusion criteria to collect human liver tissues. Total RNA was extracted from human liver tissues, and qualified RNA was reverse transcribed into cDNA and sequenced by the Beijing Institute of Genomics RNA sequencing (RNA-seq) platform (BGISEQ-500RS). Drug metabolism genes are derived from genes involved in phase I metabolic reactions, phase II metabolic reactions, and drug transport processes (1, 2). In our previous study, we conducted a comprehensive literature and National Center for Biotechnology Information (NCBI) database search based on the above classification to develop a list of 612 drug metabolism genes in mice (9). Meanwhile, in the present study, from a list of 612 drug metabolism genes, we generated a list of 476 genes present in humans and mice (see **Supplementary Material**). Then, we screened for differentially expressed genes (DEGs) in RNA-seq results based on a list of 476 drug metabolism genes. However, due to the small sample size and high false positive rate of a single transcriptome analysis, it may be difficult to obtain reliable results. Therefore, we re-analyzed RNA-seq results from our previous animal research and two GEO datasets (GSE89632 and GSE63067) by heat map, principal component analysis (PCA), and volcano map. We identified the common DEGs in the human liver samples, two GEO datasets, and our previous mouse research. Then we verified the expression of common DEGs in human samples by quantitative real-time polymerase chain reaction (qRT-PCR) to confirm our results. Finally, we performed a comprehensive literature study to generate lists of common DEG functions and substrates. The flow diagram of our study design is shown in **Figure 1**.

### 2.2 Population of the study

Between November 2020 and May 2021, NAFLD and HC patients’ liver samples were recruited from the Hospital of Zunyi Medical University. These individuals were subjected to an ultrasonography examination. Technical parameters were adjusted for each patient using this study’s standard protocol for a right upper quadrant ultrasound examination. The staff radiologists with 10 to 15 years of experience in sonography independently reviewed the images and evaluated the liver for the presence of steatosis. The liver was considered normal if the echotexture was homogeneous with no acoustic attenuation, the portal veins were visible, the diaphragm was well visualized, and the echogenicity was similar or slightly higher than the echogenicity of the renal parenchyma. The liver was characterized as fatty liver when the liver had areas of significantly increased echogenicity relative to the renal parenchyma, the ultrasound beam was attenuated with the diaphragm indistinct, or the echogenic walls of the portal veins were less visible. The severity of hepatic steatosis present by imaging was not graded (10).

Inclusion criteria were: (1) aged ≥ 18 years; (2) ultrasound evidence of a normal liver (no steatosis or cirrhosis) for HC; and (3) ultrasound evidence of fatty liver in the absence of other (viral, alcohol, metabolic) reasons for NAFLD. Exclusion criteria were as follows: (1) pregnancy or breastfeeding; (2) excessive alcohol consumption (>210 g/week in men and >140 g/week in women); (3) use of medications that may cause fatty liver (e.g., methotrexate, amiodarone, tamoxifen, and systemic corticotherapy); (4) other causes of chronic liver disease (chronic viral hepatitis B or C, hemochromatosis, autoimmune hepatitis, cholestatic liver diseases, alpha-1 antitrypsin deficiency, and Wilson disease); (5) decompensated cirrhosis (encephalopathy, variceal bleeding, liver failure, and ascites); (6) systematic infection; and (7) hepatocellular carcinoma (11, 12). During laparoscopic cholecystectomy, liver tissue was collected by a wedge liver biopsy from the free edge of the liver lobe. This study was approved by the Biomedical Research Ethics Committee of the Affiliated Hospital of Zunyi Medical University (ethics approval number: KLL-2020-046). This study was registered with the Chinese Clinical Trial Registry (registration number: ChiCTR2100041714, http://www.chictr.org.cn/). All participants provided informed written consent.

### 2.3 RNA extraction and sequencing

Total RNA was extracted from 35 human liver tissues according to the following procedures. Approximately 10–20 mg of liver tissues were homogenized in 1 mL TRIzol (Thermo Fisher Scientific, Shanghai, China) on ice, incubated for 5 min, then 200 μL chloroform was added. After low-temperature centrifugation, 500 μL isopropanol was used to precipitate RNA. The precipitate was washed with 75% ethanol (DEPC water configuration) and then dissolved in 30 μL DEPC water (Shanghai Generay Biotech Co., Ltd, Shanghai, China) after evaporating all ethanol. The RNA concentration, purity, and integrity were measured by Agilent 2100 Bioanalyzer (Agilent Technologies Inc., Beijing, China). The qualified RNA was reverse transcribed into cDNA and sequenced by BGISEQ-500RS (Beijing Genomics Institute, Beijing, China). The original data were aligned and annotated, referring to GRCh38.p12 Homo sapiens genome. And the FPKM (fragments per kilobase of exon model per million mapped reads) value was used as the standardized numerical output gene expression matrix for data analysis.

### 2.4 Gene expression omnibus datasets

The gene expression datasets re-analyzed in this study were obtained from the GEO database (https://www.ncbi.nlm.nih.gov/geo/). We selected two gene expression profiles (GSE63067, https://www.ncbi.nlm.nih.gov/geo/query/acc.cgi?acc=GSE63067, PMID: 25993042 and GSE89632, https://www.ncbi.nlm.nih.gov/geo/query/acc.cgi?acc=GSE89632, PMID: 25581263) related to Homo sapiens’ NAFLD from the database. Among them, GSE63067 was based on the Agilent GPL570 platform ([HG-U133_Plus_2] Affymetrix Human Genome U133 Plus 2.0 Array), and GSE89632 was based on platform GPL14951 (Illumina HumanHT-12 WG-DASL V4.0 R2 expression beadchip). GSE63067 (13) included 18 participants (2 SS, 9 NASH, 7 HC). Liver samples were obtained during laparoscopic bariatric surgery (for NAFLD) or laparoscopic cholecystectomy (for HC). GSE89632 (12) included 63 participants (20 SS, 19 NASH, 24 HC), and liver tissues were collected during percutaneous needle biopsy (for NAFLD) or as a wedge biopsy during hepatectomy (for HC). All of the data were freely available online.

### 2.5 Data processing

The R program was used to visualize the transcriptome levels of 476 drug metabolism genes in the liver. The heat map was generated using the “heatmap.2” function of the “gplots” package in the R program. In addition, all 476 drug metabolism gene expression values for each sample were normalized using the “scale” function of the R program. Batch correction was done *via* the “removeBatchEffect” function of the limma package in the R program. The PCA was performed using the “pca” function of the “mixOmic” package in the R program (14). And the PCA plotting of the corrected gene expression values used the “plotPCA” function. A scattered point of PCA represented a sample and included the calculated values of all 476 drug metabolism genes for each sample. We used an ellipse to encompass the scattered points corresponding to the samples in the same group. The ellipse indicated the 95% confidence interval within a group for the PCA. And scattered points and ellipses demonstrated the degree of similarity in samples within a group and the differences between the groups.

Cluster analysis, the most widely used unsupervised learning technique, is a multivariate technique for identifying subgroups in a data set with similar characteristics. In our study, agglomerative clustering algorithms were used to group samples from four merged cohorts after the batch correction. In this method, each variable is initially treated as its cluster, and then the clusters are combined hierarchically. The clusters with the shortest distances were combined first. Then, the complete-linkage function, which uses a greatest-distance metric between clusters, was then selected to perform the cluster analysis. The results were shown in a hierarchical clustering tree diagram illustrating the relationship between all samples from four merged cohorts. Hierarchical clustering of samples by all 476 drug metabolism genes was performed using hierarchical cluster function “hclust” from base package stats of R. The hierarchical clustering tree diagram is generated by the R package “ggtree” (15).

The volcano plot was carried out by R package “ggplot2”, “ggpubr”, “mixOmics”, “ggrepel”, “gplots”. The correlation and the box plot were generated by the “cor” and the “boxplot” functions in the R program. The GEO2R online analysis tool (https://www.ncbi.nlm.nih.gov/geo/geo2r/) was used to detect DEGs between NAFLD and HC samples for the GEO dataset. And the intersecting part was identified using the Venn diagram web tool (https://bioinfogp.cnb.csic.es/tools/venny/index.html).

### 2.6 The primers synthesis and quantitative real-time polymerase chain reaction

In this study, the transcriptome sequencing data were re-analyzed to screen for the DEGs. The DEGs were validated by qRT-PCR experiments to strengthen the reliability of transcriptome sequencing results. The primers were designed with Primer-BLAST online software (https://www.ncbi.nlm.nih.gov/tools/primer-blast). The primers are listed in **Table 1**. The cDNA samples were processed as recommended by Prime Script RT reagent kit (Takara, Japan) on a Mastercycler gradient PCR thermal cycler (Eppendorf Scientific, Inc., Germany), according to the manufacturer’s instructions. The qRT-PCR was performed using SYBR Green Supermix (Bio-Rad, Germany). The qRT-PCR reactions were conducted on a CFX96 qRT-PCR system (C1000 Touch, Bio-Rad, Germany). After checking the specificity of the PCR products with the melting curve, Ct values were extrapolated to a standard curve performed simultaneously with the samples, and then the data were normalized to GAPDH expression.

**Table 1:** Primers used for qRT-PCR.

Gene Name	Forward Primer	Reverse Primer
*CYP20A1*	CGATCTTCGCCGTTACCTTCT	TGCCCAAACTAACCACGAGG
*CYP2U1*	GCCTGCTGTATATGTCGCTGAAC	CCTCTGCACTTCCATGATGGTG
*SLC26A6*	CACCTCCCGGTTTTGGTCTG	CAGGCCGGATAACAGGTCAC
*SLC31A1*	CAATACAGCTGGAGAAATGGCT	TCCATTTGGTCCTGGGACAG
*SLC46A1*	AACTAAGCACACCCCTCTGC	AGGAAAAGCAACCCATATCCTGT
*SLC46A3*	AGGGTGCCGTTCCTTTTCAC	TGGTACACTTGACAACACATAGAC
*SLC9A6*	TGCCTACTGTTTGGTGCCAT	TGCCACTATTGAGGAGGACAG
*SULT1B1*	TTGAACAGTTCCATAGCAGACC	CAGGGAGAGTCATTTCCAACATT
*UGT2A3*	GCCTTCGTTAATTGACTACAGGA	GTTGATAAGCCTGGCAAGACAT
*GAPDH*	TCGGAGTCAACGGATTTGGT	TTCCCGTTCTCAGCCTTGAC

### 2.7 Statistical analysis

Variance analyses and significance tests were performed using SPSS Statistics 18.0 (IBM, Chicago, USA). Data were presented as the mean ± SD for continuous variables. All *P* values were two-sided, and p-values <0.05 were considered statistically significant.

## 3 Results

### 3.1 The baseline demographic and biochemical data

The baseline demographic and biochemical data are presented in **Table 2**. In this study, 35 human liver tissues (17 HC and 18 NAFLD) were collected by a wedge liver biopsy from the free edge of the liver lobe during laparoscopic cholecystectomy for sequencing. A total of eight (44.4%) of the patients were male. In terms of gender distribution, there was no difference between the groups. The mean body mass index (BMI = weight (kg)/[height (cm)]^2^) in the NAFLD group and HC group was 26.8 ± 3.9 and 23.3 ± 3.0 kg/m^2^, respectively. The mean alanine aminotransferase (ALT) levels were 24.2 ± 13.0 IU/L in the NAFLD group versus 19.8 ± 7.2 IU/L in the HC group. And the mean aspartate aminotransferase (AST) levels were 23.7 ± 7.4 IU/L in the NAFLD group versus 22.1 ± 4.3 IU/L in the HC group. In short, there was no difference in ALT and AST levels between the NAFLD group and the HC group. The baseline demographic and biochemical data from our previous mouse study, as well as two GEO datasets (GSE89632 and GSE63067) on the NCBI bioinformatics analysis platform, are listed in **Table 2**.

**Table 2:** The baseline demographic and biochemical data for the four cohorts.

Cohorts	Human liver samples		GSE63067		GSE89632		High-fat diet mice	
Species	Human		Human		Human		Mouse	
Group	NAFLD	HC	NAFLD	HC	NAFLD	HC	HFD	ND
n	18	17	11	7	39	24	5	5
Male, n (%)	8 (44.4)	6 (35.3)	–	–	23 (59.0)	11 (45.8)	5 (100.0)	5 (100.0)
Age, years	50.5 ± 13.0	44.1 ± 11.1	–	–	44.1 ± 10.9	37.2 ± 10.8	0.9	0.9
Height (m)	1.59 ± 0.1	1.629 ± 0.1	–	–	–	–	–	–
Weight (kg)	67.2 ± 9.0	62 ± 10.0	–	–	–	–	44.0 ± 6.2	30.3 ± 2.1
BMI (kg/m^2^)	26.8 ± 3.9	23.3 ± 3.0	–	–	30.3 ± 4.4	26.1 ± 5.6	–	–
ALT (IU/L)	24.2 ± 13.0	19.8 ± 7.2	–	–	66.5 ± 49.5	16.0 (11.0)	–	–
AST (IU/L)	23.7 ± 7.4	22.1 ± 4.3	–	–	41 ± 27.0	18.5 (7.0)	–	–
Collection time, years	2020-2021		–		2007-2011		2016	
Reference, years	–		(13), 2007		(12), 2015		(9), 2020	

### 3.2 Overall profile analysis of drug metabolism genes

**Figure 2:**
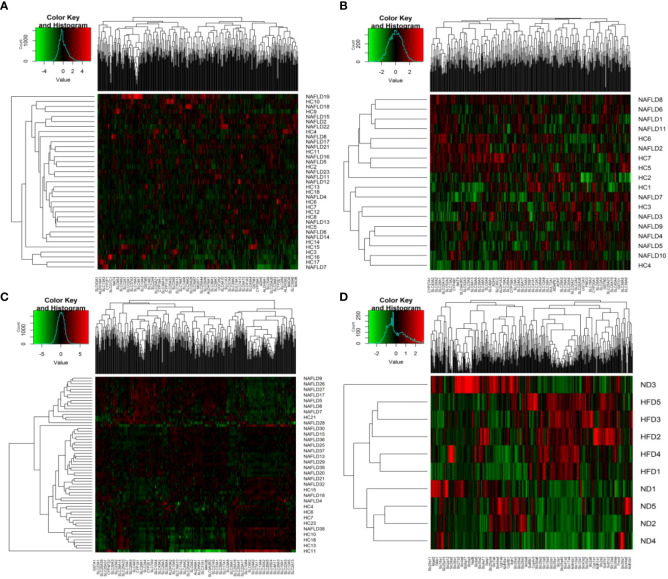
Heat map visualization. **(A–D)** Hierarchical clustering of drug metabolism genes was used by heat map. **(A–C)** were the analysis results in the human liver samples collected, the GEO dataset GSE63067, and the GEO dataset GSE89632, respectively. **(D)** belonged to the analysis results of mouse data in our previous study. NAFLD, nonalcoholic fatty liver disease; HC, healthy control; ND, normal diet; HFD, high-fat diet.

The heat map is a visualization method for analyzing the distribution of experimental data and can be used for quality control and visualization of differential data, as well as for clustering data and samples and observing sample quality. Each square indicates the relative expression of each gene in the matrix, the higher the expression the darker the red, and the lower the expression the darker the green. Each block was assigned a color according to the color change ruler (color key) the system gave. A color key shows the map between the data range and the colors. Each column indicates the gene expression in different samples, and each row indicates the expression of all genes in each sample. In **Figure 2**, there was no clear clustering in humans and GSE63067, but clear clustering in GSE89632 and high-fat diet mice. As shown in **Figure 2C**, the genes on the left displayed a similar expression pattern. These genes had higher expression in the NAFLD group. In contrast, the genes on the right displayed similar expression patterns, indicating that the HC group had higher expression levels. In **Figure 2D**, the genes on the right had higher expression levels in the high-fat diet (HFD) group. Conversely, the genes on the left had higher gene expression levels in the normal diet (ND) group.

**Figure 3:**
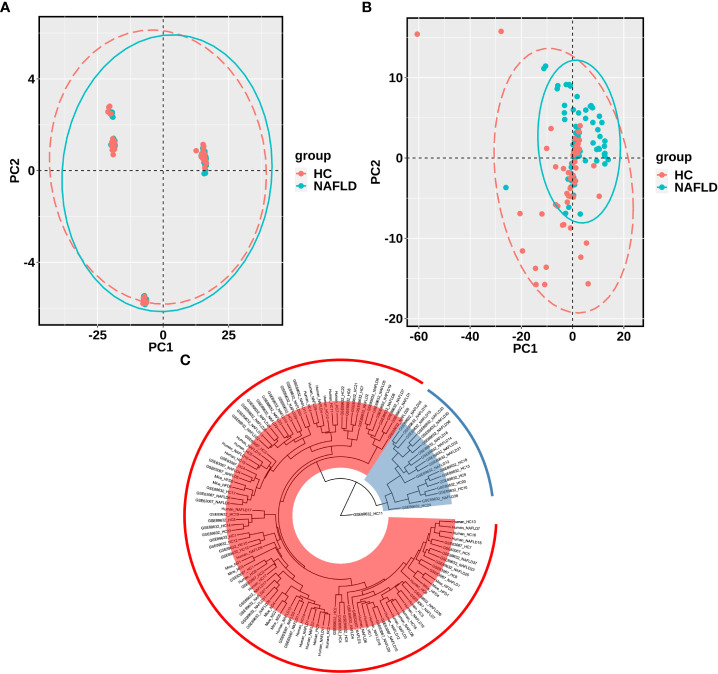
PCA and cluster analysis of the four merged samples after batch correction. **(A)** PCA before batch correction; **(B)** PCA after batch correction. **(C)** Cluster analysis of samples from the four merged after batch correction. A scattered point of PCA represented a sample and included the calculated values of all 476 drug metabolism genes for each sample. The ellipse indicated the 95% confidence interval within a group for the PCA. In A and B, the HC group was in red, and the NAFLD group was in blue. In C, Branch length represents the distance between samples, with shorter branches for more closely related samples. Samples in the same color belong to the same group. PCA, principal component analyses. NAFLD, nonalcoholic fatty liver disease; HC, healthy control.

**Figure 4:**
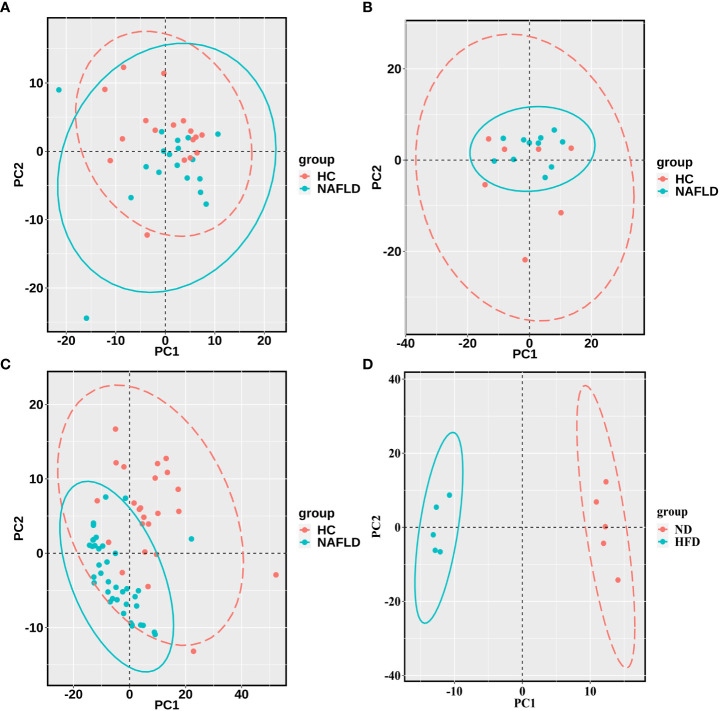
PCA for each of the four cohorts **(A–D)**. **(A–C)** were the analysis results in the human liver samples collected, the GEO dataset GSE63067, and the GEO dataset GSE89632, respectively. **(D)** belonged to the analysis results of mouse data in our previous study. A scattered point of PCA represented a sample and included the calculated values of all 476 drug metabolism genes for each sample. The ellipse indicated the 95% confidence interval within a group for the PCA. The HC groups are in red, and the NAFLD groups are blue in the human samples, while the ND groups are in red, and the HFD groups are blue in the mouse data. PCA, principal component analyses; NAFLD, nonalcoholic fatty liver disease; HC, healthy control; ND, normal diet; HFD, high-fat diet.

The sequencing data of the collected human liver samples were merged with sequencing results from the two GEO datasets and mouse data of our previous research to co-analyze by the PCA. The ellipse and the scattered points corresponding to the samples are shown in red in the HC group and blue in the NAFLD group in humans and mice. In **Figure 3A**, there was the presence of batch correction among the four cohorts. The PCA of 476 drug metabolism genes between the HC group and the NAFLD group after the batch correction is shown in **Figure 3B**. It indicated that these samples in the NAFLD group were highly similar, while the similarity in the HC group was relatively low. And the difference between the HC and NAFLD groups was not obvious. After batch correction, 126 samples from four merged cohorts were examined using cluster analysis. A sample clustering tree diagram analysis showed that the cluster was clustered into two groups (**Figure 3C**). In the first group, some samples from the GEO dataset GSE89632 were clustered together, of which 11 NAFLD samples and four HC samples were clustered into separate clusters. While the rest of the samples from the four merged cohorts were grouped in the second group. Then, we used PCA to analyze all 476 drug metabolism genes in the four cohorts, respectively. In **Figure 4**, the similarity among these samples is relatively lower in each group of the collected human liver samples and the GEO dataset GSE63067 than in each group of the GEO dataset GSE89632. Moreover, the difference in samples between groups was only apparent in our previous mouse study and the GEO dataset GSE89632.

### 3.3 Differentially expressed genes

**Figure 5:**
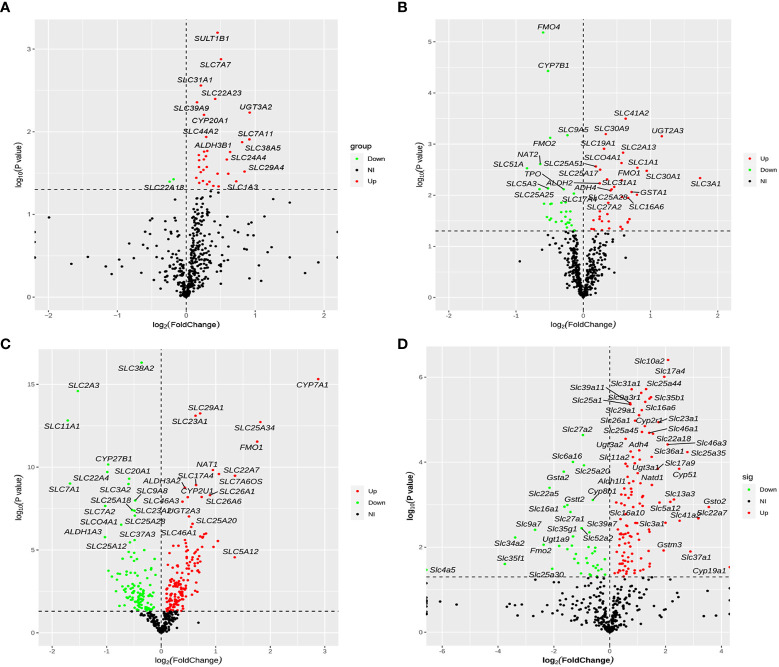
Volcano plot of differentially expressed drug metabolism genes for four cohorts. **(A–D)** The four cohorts were the human liver samples collected, the GEO dataset GSE63067, the GEO dataset GSE89632, and our previous mouse data. The screening criteria for DEGs were as follows: P-value <0.05 and |fold change|>1. Red represented up-regulated genes, green represented down-regulated genes, and black represented no significant DEGs. DEGs, differentially expressed genes.

After removing duplicate genes and expression values lacking specific gene symbols, the volcano plot based on drug metabolism gene expression data was made as shown in **Figure 5**. The screening criteria for DEGs were as follows: *P*-value <0.05 and |fold change|>1. Red represented up-regulated genes, green represented down-regulated genes, and black represented no significant DEGs. In total, 35 genes were significantly altered between NAFLD and HC groups, with 33 up-regulated genes and two down-regulated genes (**Figure 5A**). Seventy-one DEGs were found in the GSE63067 profile, including 32 down-regulated and 39 up-regulated genes (**Figure 5B**). Whereas 276 DEGs were identified in the GSE89632 profile, including 157 up-regulated and 119 down-regulated genes (**Figure 5C**). One-hundred-fifty-eight DEGs were screened (*P* < 0.05) in high-fat diet mice, with 117 genes significantly up-regulated and 41 genes significantly down-regulated (**Figure 5D**).

### 3.4 Venn diagram analysis of differentially expressed genes

**Figure 6:**
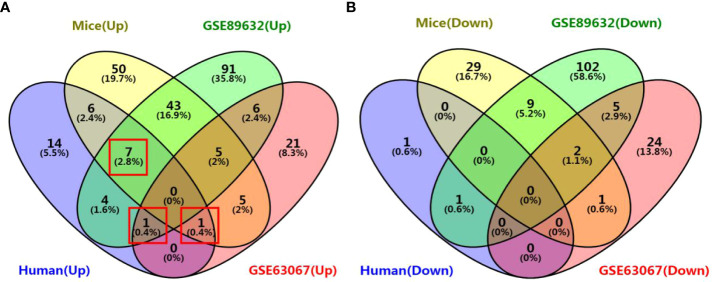
Venn diagram of common DEGs associated with drug metabolism among the four cohorts. **(A)** Venn diagram of up-regulated genes; **(B)** Venn diagram of down-regulated genes. The four cohorts were the human liver samples collected, the GEO dataset GSE63067, the GEO dataset GSE89632, and our previous mouse data.

We identified 35, 71, 276, and 158 potential DEGs in the human liver samples collected, the GEO dataset GSE63067, the GEO dataset GSE89632, and our previous mouse data, respectively (**Figures 5A–D**). The intersection of these four cohorts revealed no common DEGs. However, we identified nine common DEGs of 35 DEGs in humans, including *CYP20A1*, *CYP2U1*, *SLC9A6*, *SLC26A6*, *SLC31A1*, *SLC46A1*, *SLC46A3*, *SULT1B1*, and *UGT2A3. UGT2A3* was the only common DEG found in the human liver samples collected and two GEO datasets (GSE63067 and GSE89632). *SLC31A1* was the only common DEG found in the human liver samples collected, our previous mouse data, and the GEO dataset GSE63067. Seven genes were common in the human liver samples collected, our previous mouse data, and the GEO dataset GSE89632, including *SLC26A6*, *SLC46A1*, *CYP2U1*, *SLC9A6*, *SLC46A3*, *CYP20A1*, and *SULT1B1*, all of which were up-regulated (**Figure 6**). In addition, the functional roles and substrates of the nine common genes are shown in **Tables 3** and **4**.

**Table 3:** Functional roles of the nine common differentially expressed genes.

NO.	Gene_ID	Gene symbol	Function	Refs.
1	57404	*CYP20A1*	–	–
2	113612	*CYP2U1*	Metabolizes arachidonic acid, docosahexaenoic acid, and other long-chain fatty acids	(16, 17)
3	10479	*SLC9A6*	Sodium-hydrogen exchanger. May be involved in regulating endosomal pH and volume	(18, 19)
4	65010	*SLC26A6*	Involved in transporting chloride, bicarbonate	(20, 21)
5	1317	*SLC31A1*	High-affinity copper transporter. Functions as a homotrimer to affect the uptake of dietary copper	(22, 23)
6	113235	*SLC46A1*	A transmembrane proton-coupled folate transporter protein that facilitates the movement of folate and antifolate substrates across cell membranes	(24, 25)
7	283537	*SLC46A3*	Lysosomal copper transporter. Modulated intracellular copper levels	(26)
8	27284	*SULT1B1*	Sulphated exogenous substances involved in xenobiotic detoxification	(27)
9	79799	*UGT2A3*	Glucuronidated bile acids, particularly hyodeoxycholic acid at the 6-hydroxy position	(28)

**Table 4:** Substrates of the nine common differentially expressed genes.

NO.	Gene symbol	Drugs(substrates)	Refs.
1	*CYP20A1*	–	–
2	*CYP2U1*	Arachidonic acid, N-arachidonoylserotonin, Vitamin B2	(29, 30)
3	*SLC9A6*	–	–
4	*SLC26A6*	–	–
5	*SLC31A1*	Cisplatin, Oxaliplatin, Carboplatin	(31–33)
6	*SLC46A1*	Folates, Methotrexate, Pemetrexed	(34–36)
7	*SLC46A3*	Maytansine	(37, 38)
8	*SULT1B1*	4-Nitrophenol, Silymarin, Daphnetin, 6-Gingerol, Dotinurad, Curcuminoid, Brivanib, 6-O-desmethylnaproxen	(39–46)
9	*UGT2A3*	Hyodeoxycholic acid	(28)

### 3.5 The quantitative real-time polymerase chain reaction validation of common genes

**Figure 7:**
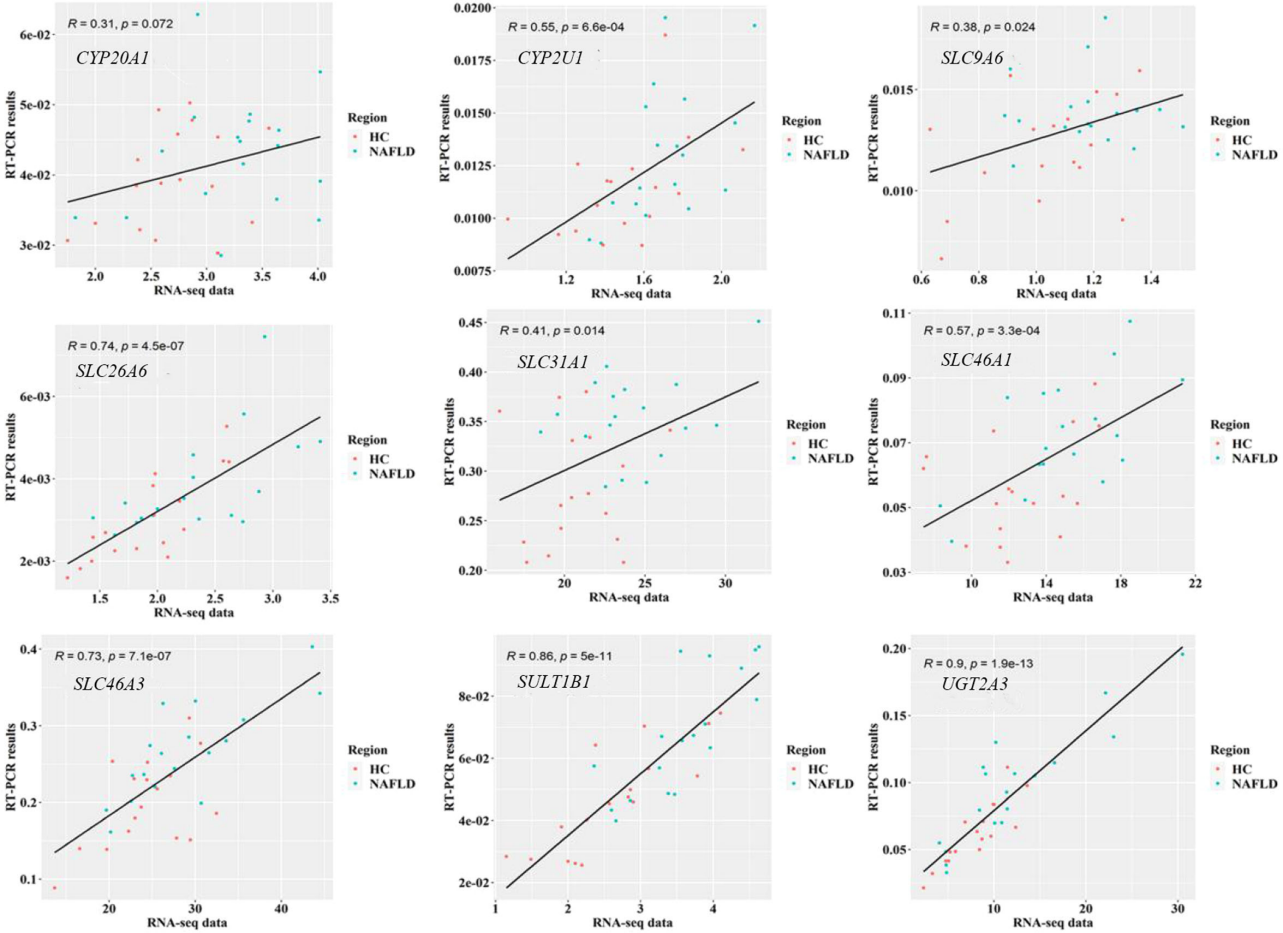
Validation of RNA-seq data on nine common DEGs in the human liver tissues using qRT-PCR technology. P<0.05 was checked by Pearson’s correlation coefficient. RNA-seq, RNA sequencing; qRT-PCR, quantitative real-time polymerase chain reaction; DEGs, differentially expressed genes.

**Figure 8:**
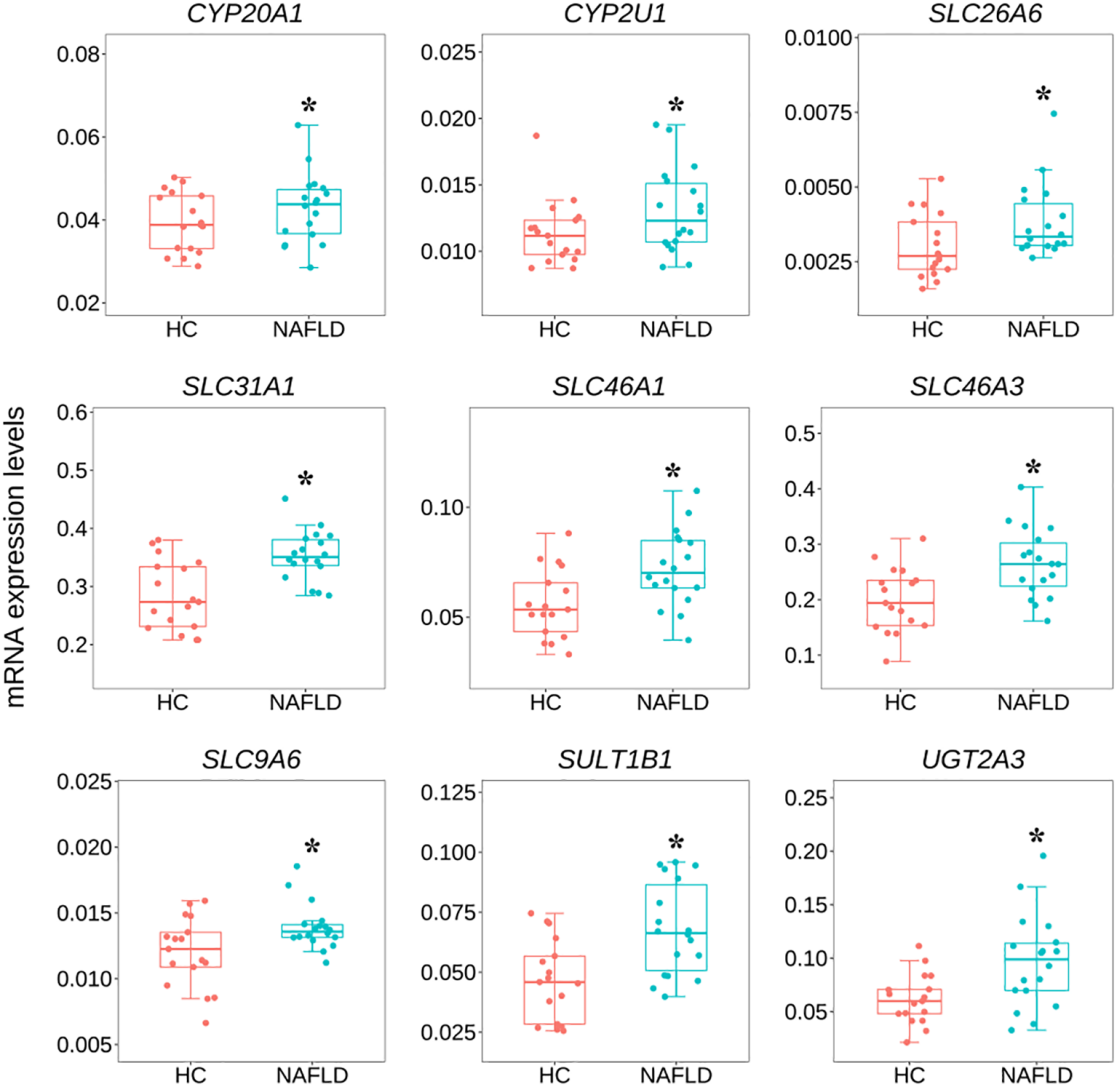
Variation of nine common DEGs between the NAFLD group and the HC group in human liver tissues by qRT-PCR. *P<0.05 was checked by the student t-test. qRT-PCR, quantitative real-time polymerase chain reaction; DEGs, differentially expressed genes; NAFLD, nonalcoholic fatty liver disease; HC, healthy control.

To validate the common DEGs at the transcriptome level in NAFLD, we compared qRT-PCR data with RNA-seq data from the same samples, including *CYP20A1*, *CYP2U1*, *SLC9A6*, *SLC26A6*, *SLC31A1*, *SLC46A1*, *SLC46A3*, *SULT1B1*, and *UGT2A3.* We noted that RNA-seq data from all nine DEGs showed a positive correlation with the data obtained by the qRT-PCR technique (**Figure 7**). In addition, the nine co-regulated DEGs were significantly up-regulated in the human liver tissues with NAFLD as shown in **Figure 8**.

## 4 Discussion

In this study, a comprehensive analysis of gene expression profiling in the liver revealed that many important drug metabolism genes were altered in NAFLD. Our present study analyzed the transcriptome of 35 human liver tissues, which contained 18 NAFLD and 17 HC liver tissues. Based on the RNA-seq data, a total of 476 drug metabolism genes were included. Our study detected only 35 DEGs between the HC group and the NAFLD group, which could be attributed to the relatively mild disease in the NAFLD group. To validate which of the 35 DEGs in humans were solid results, we examined the gene expression profiling of both GEO datasets and high-fat diet mice. Collectively, we identified nine common DEGs. Furthermore, qRT-PCR validation revealed that the expression of *CYP20A1*, *CYP2U1*, *SLC9A6*, *SLC26A6*, *SLC31A1*, *SLC46A1*, *SLC46A3*, *SULT1B1*, and *UGT2A3* were significantly up-regulated.

The PCA was used to show the degree of similarity in samples within a group and the difference in samples between the groups in the collected human liver tissues, the two GEO datasets, and our previous mouse data, based on 476 drug metabolism genes. There was the presence of batch correction among the four cohorts. And the difference in samples between the HC group and the NAFLD group was still not obvious after removing the batch correction. The merged samples were not as clearly clustered into NAFLD and HC groups as we would have preferred. So we used the PCA to analyze all 476 drug metabolism genes in the four cohorts, respectively. The difference in samples between groups was relatively obvious in our previous mouse data and the GEO dataset GSE89632. There was a discrepancy between the different studies in the PCA plots. We speculated that the reasons for the discrepancy in different human studies were as follows: different sample sizes were included in different studies; there were individual differences in human studies. Hence, the PCA plots indicated the analyzability of samples from the four cohorts, then the volcano plot was used to analyze DEGs in each cohort. A Venn diagram analysis was also used to screen for the common DEGs.

We only used RNA-seq data from drug metabolism genes in the volcano map analysis, rather than the entire dataset. As a result, the volcano plots of four cohorts swayed to the right at various levels. The volcano plot in **Figure 5A** was performed more obviously. We identified 35 DEGs in the collected human liver samples, and more DEGs were up-regulated than down-regulated just as the volcano plot displayed. Considering the volcano plot in **Figure 5A** seems swayed to the right, we screened for the DEGs based on the GEO datasets and the mouse sequencing data separately. Hence, combining with the screened results of three re-analysis studies, we identified the nine common DEGs of four cohorts to verify the reliability of this study.

In the current study, we filled some unknown fields for the SLC family in NAFLD. Our study showed that *SLC9A6*, *SLC26A6*, *SLC31A1*, *SLC46A1*, and *SLC46A3* were all up-regulated in patients with NAFLD. *SLC31A1* encodes a protein that functions as a high-affinity copper transporter in the cell membrane, regulating copper uptake and influencing dietary copper absorption (47). SLC31A1 substrates primarily include platinum antineoplastic drugs such as cisplatin (CDDP), oxaliplatin, and carboplatin (31–33). Joseph (48) et al. discovered that NASH rats given CDDP had 20% less nephrotoxicity, while hepatic CDDP accumulation was 250% higher than in healthy rats. These findings suggested that NASH-induced changes in hepatic and renal transporter expression may affect nephrotoxicity. *SLC46A1* encodes a transmembrane proton-coupled folate transporter protein that facilitates the movement of folate and antifolate substrates across cell membranes. Li (24) et al. found that *SLC46A1* could regulate iron metabolism and had a high abundance in the liver. Losing the function of the proton-coupled folate transporter protein caused hereditary folate malabsorption (49). In addition, *SLC46A1* transports folic acid, methotrexate, and pemetrexed (34–36). The lysosomal copper transporter SLC46A3 was localized to the lysosome, modulating intracellular copper levels (26). And *SLC46A3* is an effective transporter of the cytotoxic drug maytansine, which is used in antibody-based targeting cancer cells (37, 38). Moreover, we observed that *SULT1B1* was up-regulated in high-fat diet mice and patients with NAFLD. SULTs catalyze the sulfate conjugation of many hormones, neurotransmitters, drugs, and xenobiotic compounds. *SULT1B1* sulfates many exogenous substances, including 4-nitrophenol, silymarin, daphnetin, 6-gingerol, dotinurad, curcuminoid, brivanib, and 6-o-desmethylnaproxen (39–46).

When patients with pre-existing NAFLD have cancers such as lung cancer, breast cancer, and acute lymphoblastic leukemia, as well as arthritis, coagulopathy, gout, and hyperuricemia, they will be treated with cisplatin, oxaliplatin, carboplatin, pemetrexed, methotrexate, daphnetin, and dotinurad, among other drugs. Unfortunately, whether the nine common DEGs affect the pharmacokinetics (e.g., hepatic metabolism, biliary excretion, distribution, or elimination), clinical outcomes, adverse effects, and dose administration of drugs mentioned above remain unclear. The present study identified nine common DEGs in NAFLD. Although no specific recommendation is expected on dose adjustment based on small changes in drug metabolism gene expression, our research contributes to the knowledge domain associated with the transcriptomics related to NAFLD. Future research should investigate the impacts of these genes on drug dose adjustment in patients with NAFLD.

There were several limitations in our present study. First, this study had a relatively small sample size. Second, the individual sample sizes collected in this study were insufficient for Western blots and histopathology in patients with NAFLD. Third, due to a lack of detailed disease information, other diseases may regulate mRNA expression in patients with NAFLD.

## 5 Conclusions

In summary, we identified nine significant drug metabolism genes in NAFLD, including *CYP20A1*, *CYP2U1*, *SLC9A6*, *SLC26A6*, *SLC31A1*, *SLC46A1*, *SLC46A3*, *SULT1B1*, and *UGT2A3*. Future research can investigate the impacts of these genes on drug dose adjustment in patients with NAFLD.

## Data availability statement

The datasets presented in this study can be found in online repositories. The data presented in the study are deposited in the ArrayExpress repository, accession number E-MTAB-12445. The names of the repository/repositories and accession number(s) can be found in the article/**Supplementary Material**.

## Ethics statement

The studies involving human participants were reviewed and approved by Biomedical Research Ethics Committee of the Affiliated Hospital of Zunyi Medical University. The patients/participants provided their written informed consent to participate in this study. The animal study was reviewed and approved by Animal Experiment Ethics Committee of Zunyi Medical University.

## Author contributions

LiC, YH, and YW were involved in the conception and design of the study. LiC and LuC collected the samples and conducted data analysis. XL, LQ, QZ, and DT assisted in data interpretation. LC drafted the manuscript. YZ, YH, and YW critically revised the manuscript for intellectual content and approved the final version. All authors contributed to the article and approved the submitted version.

## Supplementary material

The Supplementary Material for this article can be foundonline at: https://www.frontiersin.org/articles/10.3389/fendo.2022.1034494/full#supplementary-material.
